# An Unusual Pterosaur Specimen (Pterodactyloidea, ?Azhdarchoidea) from the Early Cretaceous Romualdo Formation of Brazil, and the Evolution of the Pterodactyloid Palate

**DOI:** 10.1371/journal.pone.0050088

**Published:** 2012-11-21

**Authors:** Felipe L. Pinheiro, Cesar L. Schultz

**Affiliations:** 1 Departamento de Paleontologia e Estratigrafia, IGeo, Universidade Federal do Rio Grande do Sul. Avenida Bento Gonçalves, Porto Alegre, Brazil; 2 Bayerische Staatssammlung für Paläontologie und Geologie, Munich, Germany; Raymond M. Alf Museum of Paleontology, United States of America

## Abstract

A new and unusual specimen of a probable azhdarchoid pterosaur is described for the Early Cretaceous (Albian) Romualdo Formation of Brazil. The specimen consists of a palate that, although fragmentary, has a unique morphology differing from all other known pterosaurs with preservation of palatal elements. The new specimen probably indicates the presence of a yet undescribed pterodactyloid taxon for Romualdo Formation and brings new information on pterosaur diversity of this sedimentary unity. Mainly due to the rarity of pterodactyloid specimens with palate preservation, this structure has been overlooked in this clade. Here, we reassess the palatal anatomy of Pterodactyloidea, revealing an intriguing variety of morphotypes and evolutionary trends, some of them described here for the first time. The morphological disparity displayed by different pterodactyloid taxa may be further evidence of the presence of diverse feeding strategies within the clade.

## Introduction

The fragile nature of pterosaur skeletons has had the effect of limiting superior preservation of their remains to isolated *Lagerstätten* throughout the world [1], [2]. Even in these deposits, three-dimensional preservation rarely occurs. In most cases, pterosaur fossils are crushed, and important anatomical features are often obliterated. As a consequence, some details of pterosaur anatomy remain poorly known, which frequently leads to misinterpretations of structures. A good example of this is the pterosaur palate, because its study depends on either three-dimensionally preserved specimens or on exceptionally rare palatal views of compressed skulls. Principally because of this limitation, some bones and structures have been misidentified throughout the literature [3].

Only recently was a new interpretation of the pterosaur palate made [3], in a study that utilized the Extant Phylogenetic Bracket [4] to identify homologous structures in the palates of pterosaurs, birds and crocodiles. Although this new research, focusing on pterosaur palate anatomy, did indeed improve our understanding of this structure, it was focused primarily on non-pterodactyloids. Examining the palates of well-known pterodactyloid pterosaurs, in addition to those of still-unpublished specimens, led us to the conclusion that some anatomical features and evolutionary trends were not yet properly described for this clade. Therefore, a new examination of this subject is needed.

**Figure 1:**
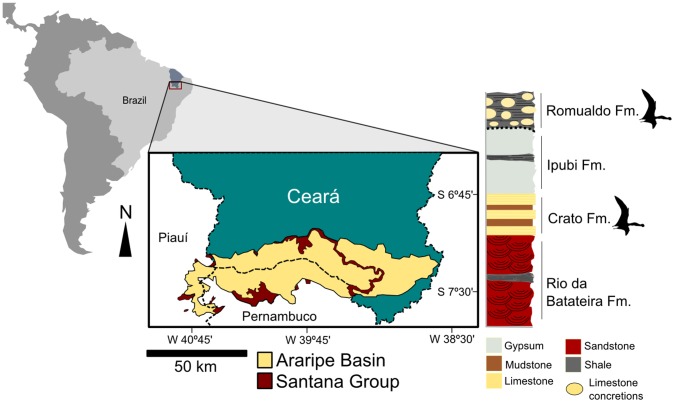
Location map of the Araripe Basin, northeastern Brazil and simplified stratigraphic chart of the Santana Group.

The Romualdo Formation of the Araripe Basin (Early Cretaceous of Northeastern Brazil) (Figure1) is probably the world’s most abundant source of three-dimensionally preserved pterosaur specimens, with some of the best pterodactyloid fossils with preservation of the palate, such as *Anhanguera blittersdorfii* [5], *A. araripensis* [6], *Tapejara wellnhoferi* [7], and *Tropeognathus mesembrinus* [8] having been found in its sediments. In fact, palatal features are often used in the diagnosis of pterosaur taxa from the Romualdo Formation, such as *Thalassodromeus sethi* [9], *Tupuxuara leonardii* [10], and *Tropeognathus mesembrinus*, among others. As will be discussed here, palatal morphology can be especially helpful in determining the taxonomy of azhdarchoid pterosaurs from this formation. The three-dimensionally preserved specimens from the Romualdo Formation, in addition to other specimens with exposed palates, can also assist in acquiring knowledge of the anatomy and evolution of this structure within the Pterodactyloidea.

We describe here a new and unusual pterodactyloid pterosaur specimen from the Romualdo Formation. The new material consists of a fragmentary palate and, although very incomplete, displays a combination of anatomical features so far never observed in other pterodactyloids. In addition, the anatomy and evolution of the pterodactyloid palate is reassessed, evidencing interesting morphologies and evolutionary trends within the clade.

## Methods

Specimen MPSC R 859 was mechanically prepared by FLP at the Vertebrate Paleontology Laboratory of Universidade Federal do Rio Grande do Sul, Porto Alegre, Brazil. Most of the other specimens analyzed and described in this paper were first hand examined by FLP, whilst other data utilized for comparisons were obtained from the literature.

Institutional AbbreviationsAMNH: American Museum of Natural History, New York, New York, USA; BSP: Bayerische Staatssammlung für Paläontologie und Geologie, Munich, Germany; DGM: Museu de Ciências da Terra, Rio de Janeiro, Brazil; IMCF: Iwaki Coal and Fossil Museum, Iwaki, Japan; KUPV: Museum of Natural History, Univesity of Kansas, Lawrence, USA; MN: Museu Nacional, Rio de Janeiro, Brazil; MPSC: Museu de Paleontologia de Santana do Cariri, Santana do Cariri, Brazil; SMNK: Staatliches Museum für Naturkunde, Karlsruhe, Germany; TMM: Texas Memorial Museum, Austin, USA; UOSG/SÃO: Collection Oberli, St. Gallen, Switzerland; YPM: Peabody Museum of Natural History, New Haven, Connecticut, USA.

## Results

### Systematic Paleontology

PTEROSAURIA Kaup 1834 [11].

PTERODACTYLOIDEA Plieninger 1901 [12].

?AZHDARCHOIDEA Nessov 1984 [13] (sensu Unwin 2003 [14]).

Gen. et sp. indet.

#### Material

**Figure 2:**
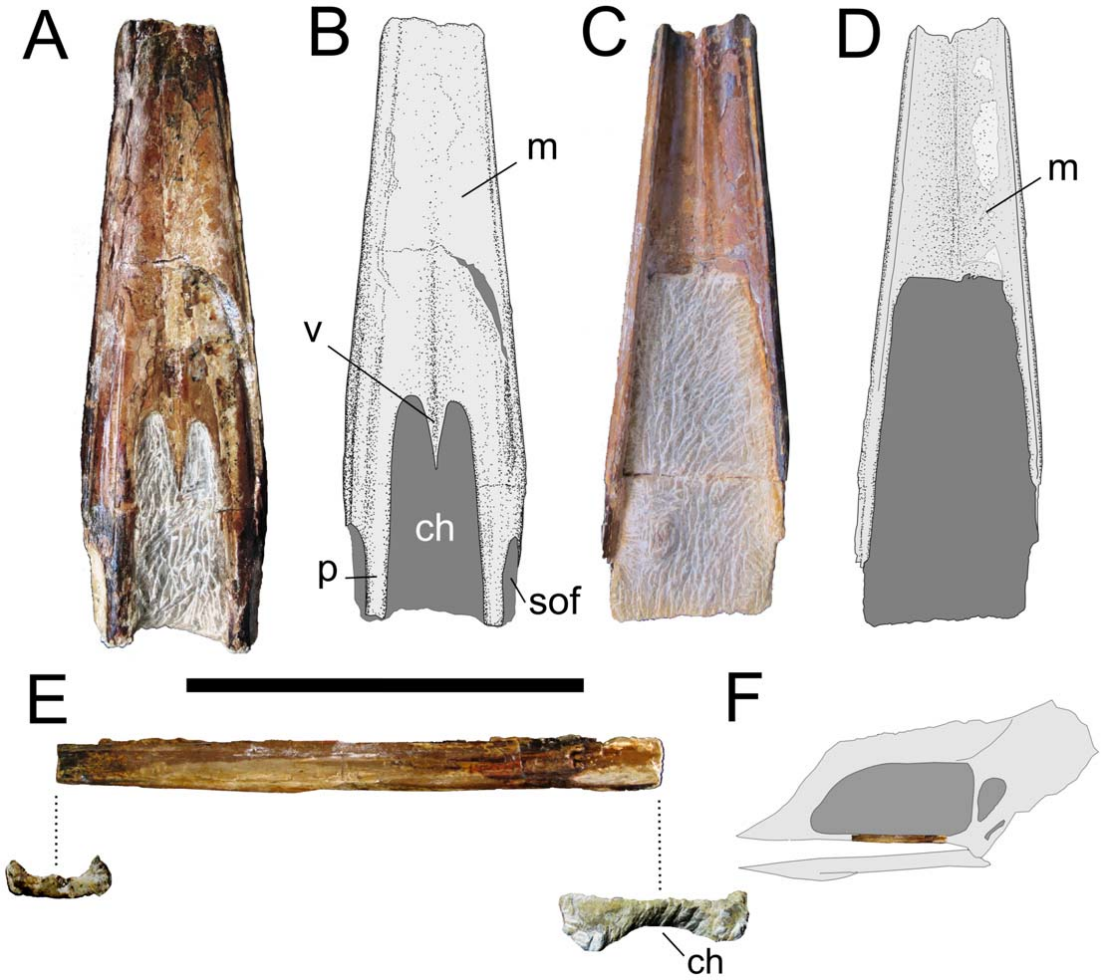
Specimen MPSC R 859 in A, B, ventral; C, D, dorsal and E, left lateral views. In F, the inferred position of the palatal fragment is demonstrated in a hypothetical azhdarchoid skull. Scale bar: 100 mm. ch, choanae; m, maxilla; p, palatine; sof, suborbital fenestra; v, vomers.

A fragmentary palate, composed mainly by the maxillae, vomers and, probably, palatines (Figure 2). The specimen is housed at the Museu de Paleontologia de Santana do Cariri (Ceará, Brazil) under the collection number MPSC R 859.

#### Locality and horizon

The specimen comes from a calcareous concretion typical of the Romualdo Formation of Araripe Basin. Nevertheless, the exact locality is unknown. The Romualdo Formation, one of the formations that compose the Santana Group, crops out throughout the Araripe Plateau, close to the boundaries of Ceará, Pernambuco and Piauí States, Northeastern Brazil (Figure 1) and is usually dated as Albian. For further information on Romualdo Formation geology, paleoecology and age, see Mabesoone and Tinoco [15], Assine [16], [17] and Martill [18].

### Description

MPSC R 859 is a fragmentary pterosaur palate, consisting of a portion of the maxillae (primarily in the form of the palatal maxillary plates), the vomers and, most likely, the palatines. The specimen has 153 mm of preserved length and 46 mm of maximum width. The straight, unbroken dorsal margins suggest that the preserved portion of the maxillae were situated under anteroposteriorly extended nasoantorbital fenestrae. Although fragmentary, the specimen is very well preserved, presenting no signs of compression. The ventral surface was exposed on the outside of the calcareous concretion and is considerably weathered. The dorsal surface was only partially prepared, because the bone becomes very thin (less than 0.5 mm thick) and fragile at the posterior half of the specimen. The choanae and the suborbital fenestrae are partially preserved. The specimen is broken approximately 97 mm from the anterior margins of the choanae, whereas posteriorly, the specimen ends 23 mm from the anterior margins of the suborbital fenestrae. Perhaps due to the weathering, the suture lines are not distinguishable in the ventral view. However, in the dorsal view, a clear medial suture separates the maxillary palatal plates.

#### Maxillae

The holotype consists almost entirely of the two maxillae. These bones are medially fused, and a clear suture line can be visualized in the dorsal view, forming a very discrete ridge. Although ventrally, there are shallow grooves between the palatal plates of the maxillae and their lateral walls, the maxillae are continuous in their dorsal aspect, with no sign of division (such as grooves or sutures) between their two distinct components. From the dorsal view, the maxillae are concave, with the palatal plates curving gently into the lateral rims. The ventral grooves between the palatal plates and the lateral walls of the maxillae, which are often visible in pterodactyloid palates, have been interpreted by most authors as being the sutures between the maxillae and the palatines (see [3] for a revision).

The palatal maxillary plates form a flat ventral surface, with no sign of palatal ridges. The palate is very slightly depressed medially, marking the place where the two maxillae fuse, although no clear sign of a suture is visible. The maxillary palatal plates are relatively thick anteriorly and gradually reduce in thickness to an exceptionally thin bony sheet in a region close to the anterior margins of the choanae. The maxillae border the choanae anterolaterally and, likely, the suborbital fenestrae anteriorly. There is no discernible suture between the maxillae and palatines.

The lateral walls of the maxillae are very slender and shallow (108 mm in height). Although subparallel posteriorly, their lateral margins begin to converge, in dorsal view, at a region close to the rostral ending of the choanae. Throughout the entire specimen, the maxillary walls constrict dorsally into very thin bony blades, which border the nasoantorbital openings ventrally. The maxillae maintain their dorsoventral height over the complete length of the specimen, with no evidence of dorsal expansion, indicating that the entire preserved portion of MPSC R 859 was located under nasoantorbital fenestrae of large proportions. Ventrally, the maxillary walls display neither teeth nor empty alveoli. The sutures between the maxillae and the vomers and between the maxillae and the palatines are not visible.

#### Vomers

The fused vomers form a slim triangular element that partially divides the choanae anteriorly. There is no sign of sutures between the two elements or between these and the maxillae. The vomers are most likely incomplete. Although elongated vomers completely dividing the choanae and contacting the medial processes of the pterygoids are visible in exceptionally well-preserved pterodactyloid specimens (e.g., *Anhanguera araripensis*), in most cases the fragility of these bones prevents complete preservation.

#### Palatines

Although these bones cannot be individualized, they likely comprise the slender components that form the margins of the suborbital fenestrae medially and the choanae laterally.

## Discussion

### Comparison and Taxonomic Assignment

As described above, the dorsal margins of the maxillae of MPSC R 859 are intact and remain straight, lacking any ascendant curvature along their entire preserved length. This indicates that all of the preserved elements were situated under nasoantorbital fenestrae of large proportions, extending well anterior of the rostral borders of the choanae.

The preserved maxillae of MPSC R 859 are edentulous throughout their length. Some toothed pterosaurs, such as members of the clades Archaeopterodactyloidea [19] (e.g., *Gnathosaurus* [20], *Feilongus* [21], *Cycnorhamphus* [22], *Ctenochasma* [23] and *Moganopterus* [24]) and Istiodactylidae [25], have teeth restricted to the anterior portion of the skull, rostral to the nasoantorbital fenestrae. However, the extension of the nasoantorbital openings in MPSC R 859 is incompatible with the clade Archaeopterodactyloidea. In the new specimen, as described above, the nasoantorbital openings extend substantially further from the anterior end of the choanae, suggesting a very large size for these fenestrae. This condition differs from the relatively short nasoantorbital openings observed in archaeopterodactyloid pterosaurs. *Moganopterus zhuiana*, referred to Boreopteridae by [24] shares some similarities with MPSC R 859. However, the two-dimensionally preserved holotype of the former prevents detailed comparisons. Although istiodactylids have exceptionally large nasoantorbital fenestrae, in these pterosaurs (at least in *Istiodactylus latidens*, the only one with three-dimensionally preserved cranial elements), the tip of the rostrum is remarkably blunt, but the maxillae converge in a higher angle than what is observed in MPSC R 859. Also, the skull of *I. latidens* is more robust, differing from the slender condition observed in the specimen we describe (see [25], [26]). Additionally, the posterior palatal anatomy of these pterosaurs remains unknown, and the extension of the choanae with respect to the nasoantorbital fenestrae cannot be determined. The palatal maxillary plates of *I. latidens*, although mainly planar, are slightly raised in a region close to the sagittal plane (Mark Witton, personal communication, 2012), also differing from the condition displayed by MPSC R 859.

While lacking elements comparable to MPSC R 859, the recently-described *Unwindia trigonus* [27], also from the Romualdo Formation, has teeth restricted to the rostral end of the skull, well anteriorly from the rostral margin of the nasoantorbital fenestrae. The incomplete nature of *Unwindia*’s holotype avoids an accurate determination of the nasoantorbital opening’s size for this taxon. Nevertheless, based on the general construction of its skull, it’s unlikely that *Unwindia* had nasoantorbital fenestrae comparable in size with what is inferred for MPSC R 859.

Because of the reasons cited above, the morphology of MPSC R 859 is more compatible with a few edentulous pterosaur taxa, so that it remains probable that the new specimen was completely toothless. Although the possibility that MPSC R 859 had teeth cannot be totally excluded, the combination of exceptionally large nasoantorbital openings, slender, anteriorly convergent maxillae and teeth restricted to the anterior end of the rostrum has never been observed in any known pterosaur taxon. Because of the probable absence of teeth in MPSC R 859, we’ll focus further comparisons of this specimen with edentulous pterosaurs (*Nyctosaurus* [28], Pteranodontidae, Tapejaridae and Azhdarchidae). However, it is worth noting that, considering the fragmentary nature of the new specimen, it is possible that more complete material of poorly known tooth-bearing taxa (such as *Unwindia* and *Moganopterus*) will, eventually, display similarities with MPSC R 859.

The palatal anatomy of *Nyctosaurus* and the pteranodontids can be reconstructed based on the few specimens preserved, at least partially, in a palatal view [28]–[31]. Both *Nyctosaurus* and *Pteranodon* [28] have comparatively short nasoantorbital fenestrae (with respect to the length of the choanae), with a very different configuration than that found in MPSC R 859, and can therefore be eliminated from the discussion. It is noteworthy that a nominal species of *Nyctosaurus* (*N. lamegoi* [32]) was proposed for the Late Cretaceous Gramame Formation of Northeastern Brazil. Nevertheless, the holotype – and only specimen known thus far – consists of a single fragmentary humerus, and its attribution to the genus can be regarded as tentative [1].

Toothless pterosaurs with proportionately large nasoantorbital openings are thus far restricted to the Azhdarchoidea (Azhdarchidae, Tapejaridae and Chaoyangopteridae *sensu* Lü et al. [33], but see [34]). Although azhdarchid pterosaurs once had a world-wide distribution, their remains are, in most cases, restricted to fragmentary postcranial bones [35]. Fairly complete skulls are known only for *Zhejiangopterus linhaiensis* [36] and *Quetzalcoatlus* [37]. Also, an incomplete skull (TMM 42489-2) from the Maastrichtian Javelina Formation (United States), sometimes attributed to Tapejaridae, may also be referred to this clade (Mark Witton, personal communication, 2012). As is common in pterosaur preservation, known *Z. linhaiensis* skulls are laterally compressed [38] and information regarding their palatal morphology is unavailable. However, this pterosaur had very large nasoantorbital fenestrae, and it is possible that the rostral margin of this opening was situated at a considerable distance from the anterior margins of the choanae. Nevertheless, direct comparisons between this species and MPSC R 859 cannot be made until more information regarding the two taxa is available.

Although badly crushed, specimens attributed to *Quetzalcoatlus* sp. with partial preservation of palatal bones were described by Kellner and Langston [39]. Similar to *Zhejiangopterus linhaiensis*, *Quetzalcoatlus* also presents large nasoantorbital fenestrae. According to Kellner and Langston [39], the choanae are incomplete in all of the specimens. However, in the best- preserved one (TMM 41961-1), these openings occupy approximately 20% of the inferred skull length. Although this measurement is an approximation, the length of the choanae with respect to the size of the nasoantorbital fenestrae in *Quetzalcoatlus* (after Kellner and Langston [39], one-third of the total skull length) seems to be incompatible with the condition observed in MPSC R 859. Nevertheless, the fragmentary nature of the latter avoids more accurate comparisons. Additionally, the palatal plates of the maxillae in *Quetzalcoatlus* (described as palatines by [39]) are flattened anteriorly, and they gradually become convex posteriorly. This contrasts with the flat maxillary plates of MPSC R 859.

The morphology of MPSC R 859 compares more favorably with that observed in members of Tapejaridae *sensu* Pinheiro et al. 2011 [34] (i.e., Tapejaridae *sensu* Kellner and Campos, 2007 [40] and Chaoyangopteridae *sensu* Lü et al. 2008 [33]). Although tapejarinid tapejarids, such as *Tapejara*, *Tupandactylus* [41] and *Sinopterus* [42], are characterized by “short-faced” skulls (at least when compared with thalassodrominid tapejarids or azhdarchids), all tapejarids have exceptionally long nasoantorbital fenestrae and lack teeth. In contrast with azhdarchids, all unambiguous species in the family Tapejaridae described thus far preserve cranial material. Additionally, Romualdo Formation Tapejaridae (*Thalassodromeus*, *Tupuxuara* and *Tapejara*) are known from three-dimensionally preserved specimens, from which palatal morphology can be assessed. Indeed, palatal characters are often used in the diagnosis of nominal tapejarid species from this formation.

Tapejarid pterosaurs have thus far been confidently recorded for the Crato and Romualdo Formations (Aptian/Albian) of the Araripe Basin (Northeastern Brazil), the Jiufotang Formation (Aptian) of Liaoning province (Northeastern China) and the La Huérguina Formation (Barremian) of Las Hoyas, Spain [34], [40], [43]. In addition, some fragmentary specimens from the Kem Kem beds (Cenomanian) of Morocco [44] and the Javelina Formation (Maastrichtian) of the United States [1], [45] may also be attributable to the Tapejaridae (but see above), indicating a worldwide distribution of this taxon during the Cretaceous.

The monophyly of Tapejaridae is still debated, with some authors supporting it [21], [34], [40], [43], [45]–[49], while others regard the taxon as paraphyletic with respect to Azhdarchidae [14], [38], [50], [51]. Although further discussions regarding the phylogeny of this taxon are beyond the scope of the present paper, a monophyletic Tapejaridae is supported herein (see [34] for a recent discussion of this issue).

The Chaoyangopteridae *sensu* Lü et al. 2008 [33] are a group of edentulous pterosaurs from the Yixian and Jiufotang Formations (Early Cretaceous of China). *Lacusovagus magnificens* [52], from the Crato Formation of the Araripe Basin, is also tentatively referred in this taxon. The taxonomic position of the Chaoyangopteridae *sensu* Lü et al. 2008 [33] is uncertain, largely due to the scarcity of information and the brief descriptions of existing specimens. Although some authors regard this taxon as closely related to the Azhdarchidae [33], [49], a recent phylogenetic analysis reclassified the group as a clade within the Tapejaridae and renamed it as Chaoyangopterinae [34]. Nevertheless, due to the scarcity of data, both positions are still disputable. In any case, all chaoyangopterinids with preserved cranial elements evidence large nasoantorbital fenestrae, in a condition similar to what is observed in other tapejarids and azhdarchoids. Unfortunately, further comparisons between MPSC R 859 and chaoyangopterinids are impossible due to the lack of preserved palatal elements in the chaoyangopterinid specimens thus far described.

Brazilian tapejarids are represented by Tupandactylus (T. imperator and T. navigans [53]) from the Crato Formation (?Aptian) and Tupuxuara (T. longicristatus [54], T. leonardii and T. deliradamus [55]), Tapejara wellnhoferi and Thalassodromeus sethi from the younger Romualdo Formation (Albian) of the Araripe Basin. As is usual in Crato Formation fossils, the specimens referred to T. imperator are laterally compressed, with no information whatsoever on palatal anatomy. The same can be stated for a number of recently described tapejarinid tapejarids from the Jiufotang Formation (northeastern China), such as Sinopterus and “Huaxiapterus” [56].

Although not mentioned in the original description of the species [53], the two specimens thus far attributed to the Brazilian tapejarinid taxon *Tupandactylus navigans* (SMNK PAL 2344 and SMNK PAL 2343) do have preserved palatal elements. Although these materials are also laterally compressed, the manner in which the bones are preserved suggests that the palate of *T. navigans* was convex in the region where the palatal openings are located.

In spite of the fact that the palatal anatomy of *Tupandactylus* remains poorly known, this genus is closely related to *Tapejara wellnhoferi*, whose palate can be assessed. *Tapejara wellnhoferi* is the best known tapejarid from the Romualdo Formation, with several specimens having been formally described [7], [57]–[59]. Most of these specimens consist of skulls with palatal components.

**Figure 3:**
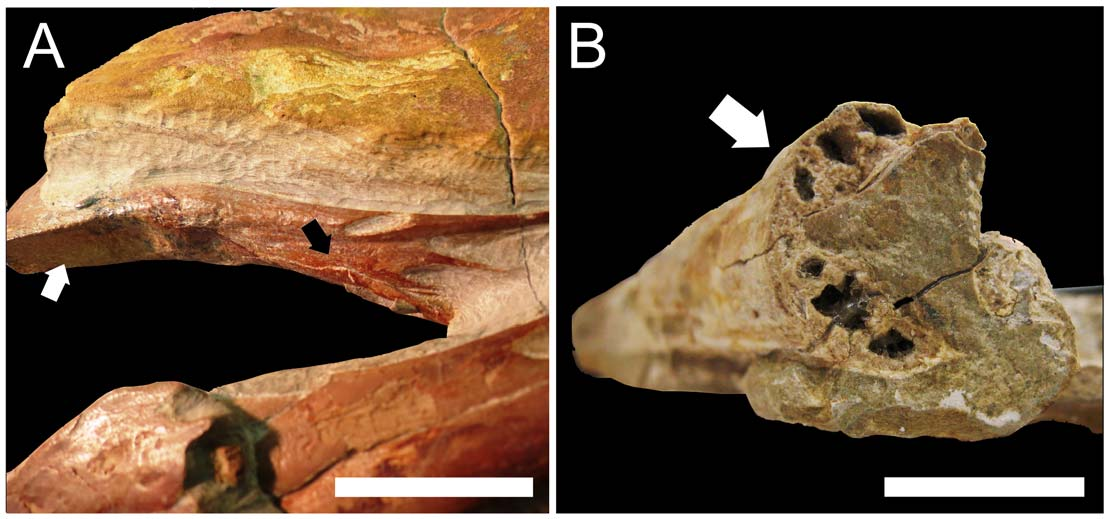
Tapejarid specimens with preservation of the palate. A, *Tapejara wellnhoferi* (AMNH 24440) showing an anterior concavity (white arrow) and a strong convexity (dark arrow) on the palatal surface; B, *Tupuxuara longicristatus* (holotype – MN 6591 V) showing the strongly convex palate that is typical of the genus. Scale bars: 30 mm in A and 20 mm in B.

As observed in the holotype (MN 6595 V) and in the specimens AMNH 2440 (Figure 3A) and UOSG 12891 that were referenced, the anteriormost region of the palatal surface of *T. wellnhoferi* bears a shallow concavity. In this region, the premaxillomaxilla is inclined downwards at an angle of approximately 25° with respect to the posterior ventral border of the maxillae [7], [57]. Following this depression, where the maxillae become abruptly horizontal, specimens AMNH 2440 and UOSG 12891 show a pronounced convexity (Figure 3A). In the holotype, this region is poorly preserved: the extremely thin bone layer collapsed, creating an artificially flat surface (FLP, personal observation). However, when the palate is intact, the choanae of *T. wellnhoferi*, closely followed by the narrow suborbital fenestrae, are located in a strong convexity, and the suborbital fenestrae can easily be observed in lateral aspect. The palatal surface of *T. wellnhoferi* bears a well-developed medial foramen, identified as a probable *foramen incisivum* by Ösi et al. [3]. Additionally, between the *foramen incisivum* and the choanae, the holotype (MN 6595 V) has two foramina that may correspond to the *aperturae maxillo-premaxillaris*. If this identification is correct, then the contact between the maxillae and the premaxillae of *T. wellnhoferi* is located in this region [3]. The palatal anatomy of *T. wellnhoferi* contrasts sharply with that observed in MPSC R 859, since the palatal surface of the latter is flat throughout its entire preserved length. Although it is possible that a convexity or concavity develops anteriorly (in the missing area of the palate), the region bearing the palatal openings is remarkably planar. Both the choanae and the suborbital fenestrae are ventrally oriented and cannot be properly seen unless in ventral aspect. Other differences between MPSC R 859 and the monospecific genus *Tapejara* include the great distance between the anterior borders of the choanae and the suborbital fenestrae (larger than the maximum width of the choanae), as well as the absence of foramina on the palatal surface in the new specimen.

The tapejarinid *Europejara olcadesorum* [43], recently described for the Lower Cretaceous of Spain, preserves some palatal elements. Nevertheless, the holotype is badly crushed and the original three-dimensional shape of the bones cannot be assessed, limiting comparisons with MPSC R 859.

The genus *Tupuxuara*, thus far composed of three nominal species (*T. longicristatus*, *T. leonardii* and *T. deliradamus*), is characterized by a strongly convex palate. As can be observed in the holotypes of *T. longicristatus* and *T. leonardii*, a median keel (which is much more developed in *T. leonardii*) originates at the anterior part of the rostrum and broadens posteriorly, where the palate becomes increasingly convex [10], [54] (Figure 3, B). The specimen IMCF 1052, illustrated in lateral aspect by Veldmeijer [60] and Witton [55], demonstrates that the palate of *T. leonardii* remains convex throughout its entire length, with the suborbital and subtemporal fenestrae being easily distinguishable in lateral view. This is also the condition described by Witton [55] for *T. deliradamus*. Although the morphology of the palate where the palatal openings are located is still unknown for *T. longicristatus*, the holotype (MN 6591 V) shows strongly convex maxillary palatal plates below the nasoantorbital fenestrae (Figure 3, B), making it likely that the condition in *T. longicristatus* was similar to that observed in other *Tupuxuara* species. Specimen MPSC R 859, therefore, differs from the genus *Tupuxuara* in having a flat palate, with no evidence of palatal ridges or convexities.

Specimen MPSC R 859 also differs from *Thalassodromeus*, the other taxon of azhdarchoid pterosaur from the Romualdo Formation. Thus far, this genus is composed of a single species, *T. sethi*, represented by the holotype (DGM 1476 R), an almost complete skull [9], and a fragmentary mandibular symphysis, which was referred (SAO 251093) [61]. *Thalassodromeus* is unique for its singular palatal configuration. The anteriormost portion of the premaxillomaxilla is convex, forming a sharp blade. Posteriorly, below the nasoantorbital fenestrae, the maxillary palatal plates become abruptly concave, with well-developed rims [9], [40], and the palate remains concave throughout its posterior length (FLP, personal observation). The absence of ventral maxillary rims in MPSC R 859 is sufficient for distinguishing the new specimen from *Thalassodromeus*.

As demonstrated above, the three genera of Romualdo Formation tapejarids can easily be distinguished from one another by their singular palatal morphologies. This strongly indicates that different feeding strategies were employed by closely related tapejarid taxa. Additionally, palatal anatomy can be a reliable source of information for the diagnosis of genera and nominal species in this clade.

As discussed, MPSC R 859 has a unique palatal morphology, different from all other pterosaurs with preserved palatal bones. Although the new specimen lacks unambiguous diagnostic characters of any known pterodactyloid clade, we tentatively attribute it to Azhdarchoidea because it probably lacks teeth and has nasoantorbital fenestrae of unusually large proportions (albeit neither of these two features are unique to the clade, this combination has thus far only been observed in azhdarchoids). A more accurate attribution of MPSC R 859 to any clade within the Azhdarchoidea is more challenging. The rarity of azhdarchid skulls, combined with the laterally compressed preservation of the majority of them, prevents a reliable reconstruction of azhdarchid palatal morphology, the same being true for chaoyangopterinids and Chinese tapejarinids. Nevertheless, as discussed above, the general construction of the *Quetzalcoatlus* skull and the inferred proportions of the nasoantorbital fenestrae with respect to the length of the choanae in this taxon is different from that found in MPSC R 859. In this respect, the new specimen is more compatible with the Tapejaridae, especially with the long-snouted thalassodrominid morphotypes.

Due to the scarcity of information concerning azhdarchid cranial morphology, it seems unwise to regard the condition observed in *Quetzalcoatlus* as the standard for Azhdarchidae, and MPSC R 859 also cannot be excluded from this clade with certainty. Nevertheless, taking into account the relative abundance of tapejarid pterosaurs in the Romualdo Formation, the absence of azhdarchids in this sedimentary unit thus far, and the general morphology of MPSC R 859 (more similar to what is currently observed in thalassodrominid tapejarids), it is also likely that the new specimen was a tapejarid.

Albeit, as discussed, MPSC R 859 has a unique palatal configuration, the new specimen is fragmentary to the extent that avoids the recognition of unambiguous diagnostic features. MPSC R 859, however, may indicate the presence of a yet undescribed azhdarchoid taxon in Romualdo Formation.

### Evolution of the Pterodactyloid Palate

As mentioned above, the study of the pterosaur palate depends upon the rare specimens in which this structure is preserved, either three-dimensionally or as an uncommon palatal view of a crushed skull. Furthermore, the high degree of bone fusion, commonly observed in pterosaur skulls, can make the delimitation of palatal elements difficult [3]. Although the absolute number of known pterosaur specimens has increased substantially during the last few decades (mainly due to the discovery of previously unknown pterosaur-bearing strata, such as the Romualdo and Crato formations in Brazil and the Jiufotang and Yixian formations in China), the relative number of skulls with preserved palates is still small. Among Pterodactyloidea, informative palatal preservation was reported or illustrated for the genera *Anhanguera*, *Ctenochasma, Dsungaripterus* [62], *Europejara, Gnathosaurus, Quetzalcoatlus, Nyctosaurus, Pteranodon, Pterodactylus* [63], *Tapejara, Thalassodromeus, Tropeognathus* and *Tupuxuara* [5]–[9], [29]–[31], [39], [43], [54], [57], [64]–[67]. See also the revision provided by Ösi et al. [3].

A major reinterpretation of pterosaur palatal anatomy was made by Ösi et al. [3] in a study that recognized crucial misinterpretations of bones and structures that were often repeated throughout the literature. The best example is the identification by most authors as “palatines” of what turned out to be palatal plates of the maxillae. The conclusions of Ösi et al. [3] are supported by topological correspondence, within an evolutionary framework provided by the Extant Phylogenetic Bracket [4]. Our observations of pterosaur specimens with preserved palates are, thus far, in agreement with the new interpretations, and the model of Ösi et al. [3] is herein supported.

Although the evolution of the pterosaur palate, culminating in the condition observed in generalized pterodactyloids, is discussed by Ösi et al. [3], this study focused primarily on non-pterodactyloid pterosaurs, especially *Dorygnathus* [68]. Our reexamination of previously described specimens, combined with published data and some as yet unpublished material, revealed that the palatal anatomy within the Pterodactyloidea is complex and cannot be generalized by a single model. Additionally, the evolution of this structure within the group shows interesting patterns, which will be discussed here.

Four major evolutionary trends were identified by Ösi et al. [3] for the palate of pterosaurs: 1) an enlargement of the choanae, following the elongation of the rostrum and the shortening of the medial processes of the pterygoids; 2) a decrease in the size of the interpterygoid vacuity; 3) an enlargement of the rostral processes of the pterygoids relative to the length of the medial processes of the same bones; and 4) a loss of the lateral processes of the pterygoids, which, in basal pterosaurs, divide the subtemporal fenestrae in two, creating the paired pterygo-ectopterygoid fenestrae. An increase in the size of the subtemporal fenestrae through their confluence with the pterygo-ectopterygoid openings would be a consequence of a more developed adductor musculature, in response to the larger jaws of pterodactyloids [3].

The assumption made by Ösi et al. [3] that their model for the palatal evolution of the Pterodactyloidea is valid for all known taxa with palatal preservation, however, proved to be false. Confluent subtemporal and pterygo-ectopterygoid fenestrae are, indeed, observable in some forms. Nevertheless, as will be demonstrated below, lateral processes of the pterygoids are secondarily developed in some taxa, while the ectopterygoids are reduced to vestigial elements in others. We describe, below, the palatal anatomy of some representative pterodactyloid pterosaurs, demonstrating the morphological diversity within the group.

**Figure 4:**
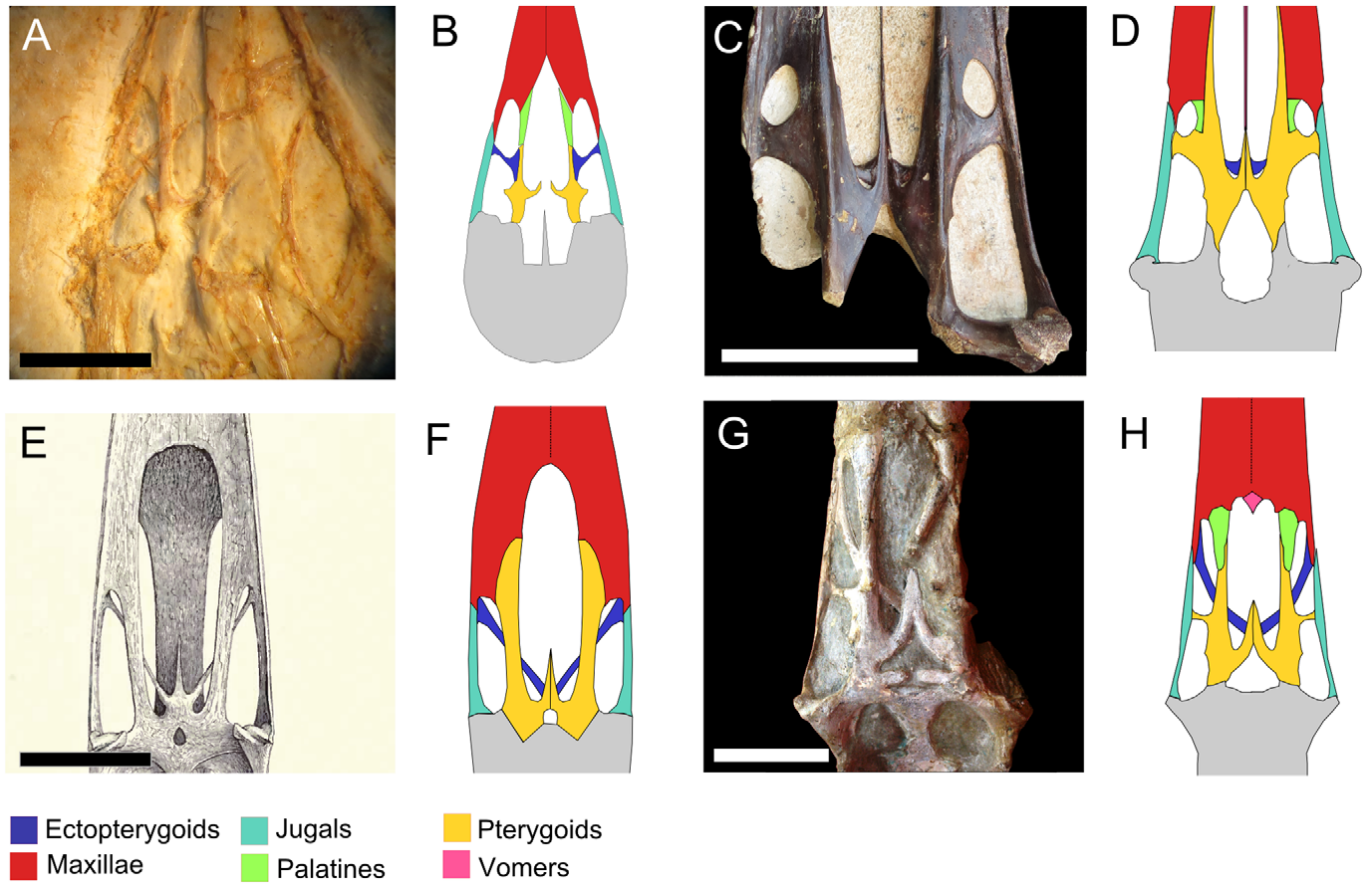
Photographs and reconstructions of representative specimens, showing palatal morphological variation among pterodactyloids. A and B, *Pterodactylus micronyx* (BSP 1936 I 50); C and D, *Anhanguera araripensis* (BSP 1982 I 89); E and F, *Pteranodon*; G and H, *Tupuxuara* (IMCF 1052). E, modified from [68]; G, photo by André Veldmeijer, courtesy of the Iwaki Coal and Fossil Museum, Japan. Scale bars: 5 mm in A and 50 mm in C, E and G.

**Figure 5:**
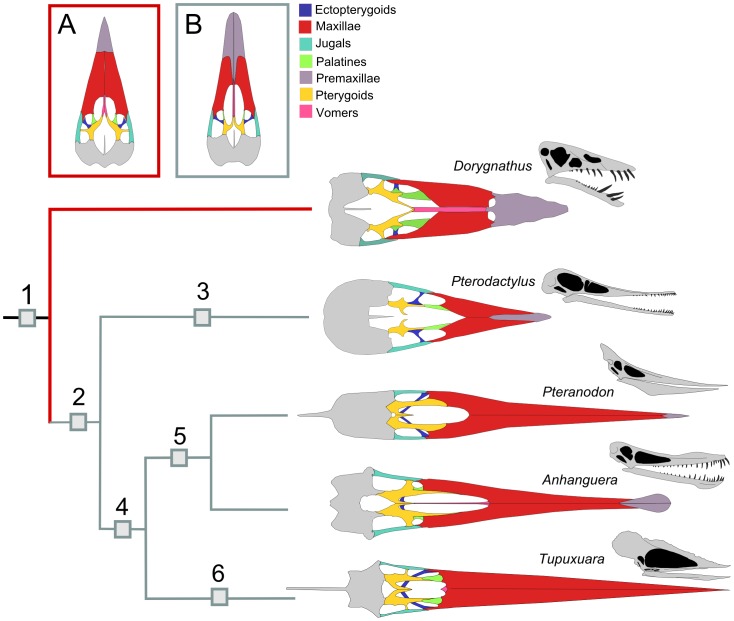
Palatal reconstructions of representative pterosaurs. A, non-pterodactyloid condition; B, primitive pterodactyloid morphology; 1, Pterosauria; 2, Pterodactyloidea; 3, Archaeopterodactyloidea; 4, Ornithocheiroidea; 5, Pteranodontoidea; 6, Tapejaroidea. A, B and *Dorygnathus* palate: redrawn from Ösi et al. [3]; *Pteranodon*: redrawn from Bennett [28]. Not to scale. The phylogenetic relationships follow the topology proposed by Kellner [46] and, more recently, Wang et al. [72].

The palate of *Pterodactylus*-like pterosaurs is, generally, inferred based on BSP 1936 I 50, a specimen attributed to “*Pterodactylus” micronyx* [69] (Figure 4A, B). The reconstruction provided by Wellnhofer [64] follows the earlier conceptualization of the pterosaur palate, with the choanae limited anteriorly by the palatines and the maxillae restricted to the dental margin. According to this author, the ectopterygoid is a well-developed element, with an anteriorly directed process that borders the choanae laterally and the “postpalatine-fenestra” medially. However, a reassessment of the specimen, under the new anatomical paradigm, showed that what was interpreted by Wellnhofer [64] as an anterior process of the ectopterygoid is, probably, the palatine. Both the choanae and the interpterygoid vacuities are relatively large and appear to be confluent, *i.e.,* the medial processes of the pterygoids do not contact each other. Nevertheless, the skull is distorted and the pterygoids are not in their natural positions, impeding an accurate estimation of the lengths of the openings. The unfused nature of the pterygoids could also be related to the ontogenetic stage of the specimen (Attila Ösi, personal communication, 2012). Interestingly, the pterygoids of “*Pterodactylus” micronyx* show laterally directed processes that do not reach the jugals, as if this taxon were transitional between a typical non-pterodactyloid palate (as seen in *Dorygnathus* and *Rhamphorhynchus* [70]) (Figure 5A) and the pterodactyloid model proposed by Ösi et al. [3] (Figure 5, B). Although the *Gnathosaurus* palate was observed only as a cast (BSP 1964 I 94), this pterosaur, closely related to *Pterodactylus*, demonstrates a similar morphology in what appears to be the primitive condition for Pterodactyloidea.

Palate preservation in *Pteranodon* is rather rare, but reconstructions were made based on specimens such as KUPV 976, 2212 and YPM 1177 [31], [71] (Figure 4E, F). This pterosaur shows a peculiar variation of the primitive pterodactyloid palate: the exceptionally well-developed ectopterygoids laterally contacted the maxillae, dorsally crossed the rostral processes of the pterygoids and contacted the fused medial processes of the latter, close to the sagittal plane of the skull. Provided that the reconstructions of Eaton [71] and Bennett [31] are accurate, there are no laterally directed processes on the pterygoids and the subtemporal openings are large. In contrast, the suborbital fenestrae are almost vestigial, constricted between the palatal plates of the maxillae anteriorly and the diagonally oriented ectopterygoids posteriorly.

An even more singular condition is observed in the Anhangueridae [5]. This taxon shares a common ancestor with *Pteranodon* at the base of the clade Pteranodontoidea [19] and is well represented by several specimens with superb palatal preservation, such as the holotypes of *Anhanguera blittersdorfii* (MN 4805 V), *Anhanguera araripensis* (BSP 1982 I 89) (Figure 4C, D) and *Tropeognathus mesembrinus* (BSP 1987 I 46). Nevertheless, the high level of bone fusion and the obliteration of the sutures in these specimens make the interpretation of the bony elements exceptionally difficult. Thus far, all anhanguerids with good palatal preservation demonstrate a small paired bony element contacting the median processes of the pterygoids laterally. These bones were ignored when *A. blittersdorffi* [5] and *T. mesembrinus* [8] were first described but were later identified as ectopterygoids in the original description of *A. araripensis* by Wellnhofer [6]. Actually, this author identifies two very distinct elements as ectopterygoids: the small bones laterally fused to the median processes of the pterygoids and the bony bridges that divide the subtemporal fenestrae from what Wellnhofer [6] called “*fenestrae postpalatinalis*”. Specimen comparisons revealed that a contact between the ectopterygoids and the median processes of the pterygoids is present in at least two other taxa of derived pterodactyloids – *Pteranodon* and *Tupuxuara.* The topological correspondence led us to conclude that the small elements described here for *Anhanguera* and *Tropeognathus* are vestigial ectopterygoids, partially agreeing with the identification by Wellnhofer [6]. Further corroboration of this hypothesis lies in the fact that, in *A. araripensis*, the distal extremities of these elements seem to lie on the dorsal surface of the rostral processes of the pterygoids, in the way that would be expected if the bridge-like ectopterygoids of *Pteranodon*, which dorsally surpass the rostral processes of the pterygoids, were reduced to their proximal ends. One implication of this interpretation is that the bony division between the lateral palatal openings is, in fact, a secondarily developed lateral process of the pterygoid (*contra* Wellnhofer [6]), and the opening identified by Wellnhofer [6] as the “*fenestra postpalatinalis*” is, in fact, a confluence between two distinct openings, topologically analogous to the suborbital and pterygo-ectopterygoid fenestrae of non-pterodactyloids (for practical reasons, we propose that this opening continues to be labeled as the suborbital fenestra in the Anhangueridae). In addition, the pterygoids are preserved in the dorsal aspect in the holotype of *Anhanguera santanae* [6], showing a continuity between the main portions of these bones and their lateral processes. Therefore, in the Anhangueridae, the “pterodactyloid model” of two paired sets of lateral fenestrae is maintained, although this is acquired by a “reversion” to a primitive condition – the presence of lateral processes on the pterygoids.

As discussed, palatal anatomy can be of special importance in the taxonomy of azhdarchoid pterosaurs, notably the tapejarids. However, in these pterosaurs, palatal characters with taxonomic relevance are thus far restricted to the region anterior to the choanae, especially with respect to the presence or absence of palatal ridges and the general morphology of the maxillary palatal plates. Few azhdarchoid specimens possess complete palates, and the posterior region of this structure is poorly known in this lineage. Nevertheless, in specimens such as IMCF 1052, attributed to *Tupuxuara leonardii* (illustrated by Veldmeijer [60] and Witton [55]), the palatal morphology can be fully assessed. IMCF 1052 displays three pairs of lateral palatal fenestrae, in a pattern that closely resembles the non-pterodactyloid condition (Figure 4G, H). As in *Pteranodon,* the ectopterygoids are exceptionally well-developed and contact the fused medial processes of the pterygoids, crossing the rostral rami of the pterygoids dorsally. Additionally, lateral processes are present on the pterygoids, dividing the subtemporal fenestrae and creating secondary pterygo-ectopterygoid fenestrae.

Although it would be unwise to state that the highly specialized morphology observed in *Tupuxuara* is the standard for the Azhdarchoidea, this genus shares some similarities with what was described by Kellner and Langston [39] for *Quetzalcoatlus*, indicating that, for some aspects, a certain level of conservativeness should be expected. As in *Tupuxuara*, Q*uetzalcoatlus* shows lateral processes on the pterygoids that, as observed by Kellner and Langston [39], probably divided the subtemporal fenestrae. In the same way, according to the authors, the ectopterygoid of *Quetzalcoatlus*, although incomplete on the specimen studied, extends diagonally above the pterygoid. Thus, it is likely that the conditions in this azhdarchid and in *Tupuxuara* were similar. Despite the fact that the palate of *Thalassodromeus sethi* was only superficially described by Kellner and Campos [9], this pterosaur also had three pairs of lateral palatal fenestrae, although the ectopterygoids are much broader and it is unlikely that they reached the median processes of the pterygoids (FLP, personal observation). The condition in *Tapejara* is currently unknown.

Piscivory is generally assumed to have been the feeding habit for most pterosaurs [1], [73], and it is indeed likely that a large number of known taxa preyed on fishes. As a matter of fact, most of the taxa studied directly herein are thought to be, at least partially, piscivorous [1], [8], [9], [31], [74]. However, studies of pterosaur feeding strategies are scarce, and conclusions are often based on superficial anatomical observations rather than on comprehensive studies of functional morphology. It is also important to observe that our knowledge of pterosaurs is remarkably biased by a “*Lagerstätten* effect”: preservation of their remains often depends upon special environmental conditions, and our understanding of pterosaur diversity is probably strongly influenced by a concentration of informative specimens in a few deposits [2], [75], [76].

The study of palatal anatomy, as well as other aspects of the feeding apparatus, is of great relevance for a better understanding of pterosaur feeding habits. The diversity of palatal morphologies described, for the first time, herein may suggest that pterodactyloids displayed complex and diversified feeding strategies, in a way analogous with was already proposed for non-pterodactyloid stem-groups [77]. The anatomical disparity between supposedly piscivorous forms, demonstrated here by several different palatal morphologies, could be evidence that piscivory emerged secondarily in a number of lineages. Nevertheless, more data are needed to test this hypothesis, mainly because, as highlighted above, most inferences about pterosaur feeding strategies are based on superficial anatomical characters. Regardless, it is likely that pterodactyloid feeding habits were much more diverse than the fossil record has led us to believe.

### Conclusions

Fragmentary remains can, sometimes, provide relevant information about fossil taxa. MPSC R 859, although very incomplete, has a unique morphology and increases our knowledge of the Romualdo Formation pterosaur fauna. The new specimen makes it clear that palatal features can be of great relevance in diagnosing azhdarchoid pterosaurs and that the variation is probably related to the development of a diversity of feeding habits among the members of this clade.

Mainly because palatal anatomy is difficult to assess in most pterosaur specimens, this region has been overlooked, with very few palatal characters being used in pterosaur phylogenetic analyses. However, as demonstrated here, the Pterodactyloidea show considerable variation in palatal morphotypes, and this diversity seems to be congruent with the proposed phylogenetic relationships of the clade. Thus, with more information available, palatal anatomy can be significant for achieving a better resolution of pterosaur phylogeny. In the same way, a better understanding of pterodactyloid palatal anatomy is of crucial relevance in accessing the feeding habits and, in a broader sense, the ecology of this clade.
